# Autonomic Stimulation Action of EAT (Epipharyngeal Abrasive Therapy) on Chronic Epipharyngitis

**DOI:** 10.7759/cureus.63182

**Published:** 2024-06-26

**Authors:** Ito Hirobumi

**Affiliations:** 1 Otolaryngology, Ito ENT Clinic, Funabashi, JPN

**Keywords:** reilly phenomenon, baroreceptor reflex (br）, epipharyngeal abrasive therapy (eat), polyvagal theory, analysis of heart rate variability (hrv), immune system stimulation, endocrine reflex, pharyngeal reflex, autonomic stimulation, chronic epipharyngitis

## Abstract

This study investigated the pathogenesis and pathophysiology of chronic epipharyngitis, which presents a variety of symptoms, with a focus on autonomic neuropathy symptoms, and also investigated the literature for information on EAT, which is useful as a treatment method. The mechanism of action of EAT has recently been clarified in terms of its immune system-stimulating and endocrine system-stimulating effects. However, the autonomic nerve-stimulating effects of EAT are still largely unexplained. This study was conducted to collect and integrate previous studies and papers focusing on the autonomic nerve-stimulating effects of EAT and to provide insight into the still not fully elucidated autonomic nerve-stimulating effects of EAT on chronic epipharyngitis. The local stimulating effects of zinc chloride and the bleeding and pain effects of EAT are also summarized, suggesting that EAT exerts its therapeutic effects through the interaction of the immune system, the endocrine system, and the autonomic nervous system. It is important to determine which mechanism is predominantly involved in each case of chronic epipharyngitis and to utilize it in treatment. Elucidating the effects of EAT on the autonomic nervous system will be an important guideline in determining the treatment strategy for chronic epipharyngitis.

## Introduction and background

B-spot therapy is a treatment method for chronic epipharyngealngitis in which nasal abrasion therapy is performed with a Lutze-type cotton swab soaked in 0.5-1% zinc chloride solution, followed by oral abrasion therapy with a Hartmann's pharyngeal wound cotton swab, and zinc chloride solution is applied to the epipharyngeal mucosa. The name B-spot therapy was proposed by Horiguchi. This is a treatment for chronic epipharyngitis developed in Japan. The diagnosis and treatment of chronic epipharyngitis were vigorously pursued in the 1960s and 1970s by Shunzo Yamazaki, the first professor of otolaryngology at Osaka Medical College, and Shinsaku Horiguchi, the first professor of otolaryngology at Tokyo Medical and Dental University [1,2]. However, the concepts of diagnosis and treatment of chronic epipharyngitis did not penetrate otolaryngologists. About 30 years later, Tanaka proposed epipharyngeal abrasive therapy (EAT), in which B-spot therapy is performed endoscopically, to improve the diagnosis and effectiveness of treatment of chronic epipharyngitis [3]. Chronic epipharyngitis is thought to be involved in a wide variety of diseases and symptoms. The diseases can be categorized by pathophysiology as follows: first, posterior rhinorrhea, sore throat, abnormal pharyngeal sensation, headache, stiff shoulders, chronic cough, and other related symptoms due to local inflammation of the nasopharynx itself [4]; second, immune diseases and related symptoms via autoimmune mechanisms such as IgA nephropathy, IgA vasculitis, nephrotic syndrome, palmoplantar pustulosis, arthritis, and sternoclavicular joint hyperplasia [5]; third, autonomic nervous system disorders such as general malaise, chronic fatigue, vertigo, sleep disorders, orthostatic dysregulation, functional gastrointestinal disorders, irritable bowel syndrome, poor memory and concentration, diseases due to endocrine system disorders, and systemic symptoms [4]. The three major groups are as follows. Chronic epipharyngitis is thought to be a combination of these conditions and to manifest a variety of symptoms (Table 1).

**Table 1:** Classification of the pathophysiology of chronic epipharyngitis Symptoms of local inflammation of the epipharynx include posterior rhinorrhea, sore throat, radiating pain, headache, and abnormal pharyngeal sensation. Autoimmune and autoinflammatory diseases associated with chronic epipharyngitis are thought to be caused by the same immunologic mechanisms involved in tonsillar lesion disease. Diseases include IgA nephropathy, palmoplantar pustulosis, arthritis, and collagen diseases. Symptoms of endocrine hormone disorders include endocrine dysfunction, adrenal fatigue syndrome, and chronic fatigue. Symptoms and diseases caused by autonomic nervous system disorders include general malaise, sleep disorders, poor memory and concentration, orthostatic dysregulation, and functional gastrointestinal disorders. Chronic epipharyngitis is thought to be a combination of these conditions and to produce a variety of symptoms. Table Credit: Author

Classification of Causes of Chronic Epipharyngitis					Main Symptoms and Diseases			
Caused by Local Inflammation of the Epipharynx					Posterior Rhinorrhea			
					Sore Throat			
					Radiating Pain			
					Headache			
					Abnormal Sensation in The Pharynx			
Caused by Immune System Disorders					Focal Infection			
					IgA Nephropathy			
					Palmoplantar Pustulosis			
					Arthritis			
					Collagen Disease			
Caused by Endocrine System Disorders					Endocrine Dysfunction			
					Adrenal Fatigue Syndrome			
					Chronic Fatigue			
Caused by Autonomic Nervous System Disorders					General Malaise			
					Sleep Disorders			
					Impaired Memory and Concentration			
					Orthostatic Dysregulation			
					Functional Gastrointestinal Disorder			

With regard to the mechanism of manifestation of symptoms due to chronic epipharyngitis, the first local symptoms may be due to direct pain from inflammation of the nasopharynx, radiating pain, or irritation of purulent rhinorrhea [4]. The second autoimmune and autoinflammatory diseases are thought to involve the same immunologic mechanisms as tonsillar focal disease, as the epipharynx, together with the palatine tonsils, constitutes Waldeyer's tonsillar ring [5-6]. The third type of symptoms caused by autonomic and endocrine disorders may be due to chronic stimulation of the autonomic nervous system due to inflammation of the epipharyngeal mucosa, venous congestion, and autonomic dysfunction due to impaired circulation in the brainstem, thalamus, and hypothalamus caused by cerebrospinal fluid congestion [4]. Chronic stimulation of the autonomic nervous system and persistent stimulation by inflammation-related factors can induce autonomic overstimulation syndrome (Reilly phenomenon) [7] by causing microcirculatory disturbances and hemorrhagic lesions [8] due to ischemia and reperfusion disorders in various organs of the body. Stress reactions based on the polyvagal theory [9] may trigger a variety of systemic symptoms. Table 2 shows a possible mechanism of autonomic symptoms caused by chronic epipharyngitis.

**Table 2:** Mechanism of autonomic symptoms caused by chronic epipharyngitis Decreased autonomic function due to irritative symptoms and persistent inflammation of the autonomic nervous system caused by persistent inflammation of the epipharyngeal mucosa. Decreased autonomic nerve center function due to impaired circulation in the brainstem, thalamus, and hypothalamus caused by epipharyngeal vein congestion, lymphatic congestion, and cerebrospinal fluid circulation disorders. Effects on various organs throughout the body due to autonomic hyperstimulation syndrome (Reilly phenomenon) caused by chronic stimulation of the autonomic nervous system and inflammation-related factors. Effects of stress reactions on the parasympathetic and sympathetic nervous systems due to the Polyvagal Theory. Endocrine and immune system abnormalities can also cause autonomic nervous system abnormalities. The combination of these causes is thought to be responsible for the development of autonomic symptoms. Table Credit: Author

Mechanisms of Autonomic Symptoms			
1. Chronic Inflammation			
2. Circulatory Disturbances			
3. Reilly Phenomenon			
4. Stress Reactions			
5. Endocrine System Disorders			
6. Immune System Disorders			

^_67_^Ga scintigraphy shows an accumulation in the epipharynx even in healthy subjects, suggesting that the epipharynx is in a state of physiologic inflammation. There are cases of subclinical chronic epipharyngitis in which local findings in the epipharynx are poor and patients have few local symptoms, despite the presence of secondary disease that may be caused by chronic epipharyngitis. Sugita reported that the diagnosis of chronic epipharyngitis and secondary disease is difficult without suspecting the possibility of chronic epipharyngitis [10]. Reasons why the concept of diagnosis and treatment of chronic epipharyngitis did not become widespread until recently include the lack of standardization of diagnosis due to the underdevelopment of endoscopic diagnostic techniques, the fact that the effectiveness of treatment varied depending on the EAT technique and skill of the surgeon, the fact that although it is a simple and useful treatment method, it is somewhat unrepeatable and objective, and the fact that it is not always easy to perform. Although it is a simple and useful treatment, it lacks reproducibility and objectivity; otolaryngologists have become skeptical of reports that EAT is effective for many diseases of unknown cause [11]; and there has been insufficient reporting and collection of evidence for chronic epipharyngitis and EAT. Currently, evidence-based chronic epipharyngitis and EAT guidelines are being developed by the Committee on Epipharyngeal Abrasive Therapy of the Japanese Society of Oral and Pharyngological Sciences.

In recent years, the pathogenesis of chronic epipharyngitis and the mechanism of action of EAT have been increasingly elucidated by immunological methods [12-13], but there are still many unexplained aspects in relation to the autonomic nervous system. The purpose of this study was to collect and integrate previous studies and papers on the pathogenesis of chronic epipharyngitis and the mechanism of action of EAT by autonomic nerve stimulation and to provide insight into the still not fully understood autonomic nerve-stimulating effects of EAT on chronic epipharyngitis. This paper is reported as a discussion for the future dissemination and development of EAT. This paper is also intended to contribute to the ongoing development of guidelines by the Committee on Epipharyngeal Abrasive Therapy

Methods

The purpose of this review was to collect previous studies on the autonomic stimulatory effects of EAT, to survey the information, and to accumulate and integrate new knowledge into the existing body of knowledge. In order to provide an overview of the therapeutic effects of EAT on chronic epipharyngitis, this review also summarizes the literature focusing on the endocrine and immune system stimulating effects of EAT to further discuss their relevance and interaction with the autonomic nerve stimulating effects. Why zinc chloride is used in EAT, the significance of EAT-induced bleeding, and the significance of pain stimulation by EAT were also discussed as subthemes. The search focused on open access publications, peer-reviewed journals, and related books, as there are few previous papers on the autonomic nerve-stimulating effects of EAT, and the majority of papers were written in Japanese, as EAT is a treatment developed in Japan. The search scoured reliable academic sources such as PubMed, Scopus, Google Scholar, and others to locate studies published in English. The academic information source J-STAGE was also scrutinized to search for papers published in Japanese.

## Review

Autonomic nerve-stimulating effect of zinc chloride in EAT

Zinc chloride (ZnCl_2_) used in the treatment is a chloride of zinc, synthesized by Johann Rudolf Glauber in Germany in 1648. It is mainly used for surface cleaning of plating and as an antiseptic, but it also denatures proteins and astringent to tissues and blood vessels [14]. Horiguchi used a 1% zinc chloride solution for the purpose of quenching the mild corrosive effect of heavy metal salts [2].

If the main mechanism of action of EAT is the anti-inflammatory effect of zinc chloride, it is thought that simply applying or rubbing a zinc chloride solution to the epipharyngeal mucosa would have a reproducible therapeutic effect. However, application and rubbing with saline solution, magnesium chloride, or other agents can similarly improve symptoms [15]. Kimura reported that the efficacy of physical epipharyngeal abrasion without the use of a chemical solution was equivalent to that of zinc chloride, and the mechanism of action was improvement of circulatory disturbance caused by physical stimulation [16]. These findings suggest that the direct anti-inflammatory action of zinc chloride may be important in the development of the effect of EAT with zinc chloride, but the immunological and autonomic nerve-stimulating effects of physical stimulation of the abrasive stimulus may also be important.

Effects of EAT-induced hemorrhage on the autonomic nervous system

Chronic epipharyngitis is thought to cause not only immune system abnormalities due to exogenous adjuvants such as bacteria, viruses, and dust, but also circulatory system abnormalities such as microvessels and capillary lymphatics, autonomic nervous system abnormalities such as vagus nerve, and endocrine system abnormalities. Vascular endothelial cells that are metabolically impaired by venous congestion produce and release inflammatory mediators, resulting in increased vascular permeability, leaky hemorrhage, and exudate leakage. On the other hand, activated lymphocytes release various inflammatory cytokines. The capillary lymphatic dilatation effect of these inflammation-related factors causes venous and lymphatic congestion and submucosal edema in chronic epipharyngitis [5].

As a mechanism for excreting cerebral waste, there is a cerebral lymphatic drainage channel called the glymphatic system, in which the perivascular lumen around cerebral arteries functions as a functional lymphatic vessel. Cerebrospinal fluid drains extracranially via the basilar region lymph vessels, spinal epidural lymph vessels, and dural lymph vessels and then passes through the pharyngeal lymph vessels to the deep cervical lymph nodes. Cerebrospinal fluid dynamics are regulated by the glymphatic system, but the circulation pathway of the nasopharyngeal cavity is important as a drainage route for cerebral waste products [17]. In chronic epipharyngitis, these cerebral lymphatic waste excretion systems are thought to be impaired.

In the early stages of treatment with EAT, bleeding during abrasion is often observed. This is because the mechanical abrasion stimulus from EAT induces bleeding from the small venous plexus of the epipharyngeal mucosa. Bleeding reduces venous and lymphatic congestion and restores cerebral venous and cerebral lymphatic excretory system function. Kuroiwa reported that the hypothalamus and periventricular organs are the security gates of the stress center and that the cerebral venous and cerebral lymphatic excretory systems are important [18]. EAT also directly improves inflammation of the local mucosa and resolves the autonomic nervous system stimulation state. It is also thought to have an autonomic nerve-stimulating effect that restores the function of the cerebral venous and lymphatic excretory systems by physically abrasive bleeding of the pathological epipharyngeal mucosa and improves autonomic nervous system function in the brainstem, thalamus, and hypothalamus.

The amount of bleeding at the time of abrasion is limited to that on a cotton swab or crimp, and the amount of bleeding on the day after EAT is also small. Even this small amount of bleeding often results in immediate improvement in clarity of vision and a feeling of heaviness in the head. It is thought that the bleeding effect is not due to the local excretion of toxins by EAT, but rather an immediate autonomic nerve-stimulating effect by improving venous and lymphatic congestion, thereby stimulating the circulatory system.

The vertebral venous plexus in the spinal canal, called Batson's venous plexus, is continuous with the venous plexus outside the spinal canal via the guiding veins. It has few venous valves and is prone to congestion. When the vertebral venous plexus outside the spinal canal, such as in the axial vertebra, becomes congested, the spinal nerves are compressed and symptoms such as headache, stiff shoulders, and back pain occur. The sympathetic ducts in the spinal column also suffer circulatory disturbance due to congestion of the venous plexus outside the spinal canal, causing pain in areas along the spinal column and visceral discomfort [19]. Congestion of the venous plexus and cavernous sinuses at the base of the brain causes circulatory disturbances in the autonomic centers of the brainstem, thalamus, and hypothalamus, which have venous and lymphatic systems flowing into those venous sinuses, resulting in the development of autonomic neuropathy symptoms. It is thought that EAT-induced hemorrhage improves venous and lymphatic system congestion, improving cerebral circulation and cerebral-lymphatic waste excretion system, thereby improving autonomic nerve sting function.

Alteration of pain sensation by EAT

Abrasive stimuli from the nasal cavity are nociceptive stimuli to the nasal cavity and epipharyngeal mucosa tissue. Fine myelinated A-δ fibers are receptive to mechanical nociception, and unmyelinated C-fiber somatosensory nerve endings are receptive to various nociceptive stimuli, including mechanical, thermal, and chemical nociception. These are called polymodal nociceptors and increase their activity in an intensity-dependent manner. The epipharyngeal mucosa is a site where the peripheral vagal afferent tract projects and is rich in unmyelinated C fibers [20]. Abrasive stimulation with EAT using zinc chloride is thought to form a somatic-visceral reflex in which information from polymodal nociceptors innervated mainly by C fibers serves as afferent input and is expressed in effectors via reflex centers and autonomic nervous system centers.

Acute pain from EAT can be classified into two types: first pain, a fast pricking pain that travels up the myelinated A-δ fibers of the peripheral nerve, and second pain, a slow burning pain that travels up the unmyelinated C fibers. When multiple fibers, A-delta and C fibers, are stimulated simultaneously, first pain from A-delta fibers is initially preferentially transmitted as sensory nerve input, but as inflammatory symptoms improve, second pain information from C fibers, which are non-nociceptive afferent fibers with a low threshold, is preferentially transmitted [21]. Although pain is intense in the early stages of EAT treatment, the nature of the pain may change as the inflammation of chronic epipharyngitis disappears.

Autonomic nerve-stimulating effects of EAT

A few papers have reported on the autonomic nerve-stimulating effects of EAT using the vasomotor reflex. Vasomotor reflexes include the vasoconstrictor reflex and the vasodilator reflex [22] and are also affected by the hardness and elasticity of the vessel wall [23]. The pneumatic strain gauge plethysmograph (PLG) was first reported by Brodie in 1905 and captures the pulsatile vasomotor reflex induced by local stimulation as a change in the volume of the pulsatile area. Harada analyzed the phalangeal vasomotor reflex during intranasal EAT. First, EAT elicited a vasoconstrictor reflex due to increased sympathetic activity, and then a vasodilator reflex due to suppressed sympathetic activity. Patients with autonomic dysregulation such as orthostatic dysregulation (OD) and vertigo had prolonged vasomotor reflex induced by EAT. EAT for these patients was reported to improve autonomic symptoms and restore the vasomotor reflexes. However, recovery of PLGs varied from case to case, and some patients required long-term treatment even after local inflammatory findings improved. In this paper, abnormal vasomotor reflexes were observed in chronic epipharyngitis, and the vasomotor reflexes were improved by EAT, suggesting that EAT has the ability to improve autonomic nervous system function [24]. The improvement of vasomotor reflexes is thought to be due to the improvement of sympathetic and parasympathetic reflexes.

Katori performed a pharmacological autonomic function test using intramuscular methacholine chloride injection while recording an otorrhea vascular volume pulsogram in a case of chronic epipharyngitis. Symptom classification of chronic epipharyngitis was performed, and the autonomic function of each case was discussed. Chronic epipharyngitis was found to be a state of subclinical persistent autonomic stimulation, with a mixture of parasympathetic and sympathetic predominant cases. The dizziness type was characterized by decreased parasympathetic and increased sympathetic function in most cases, but there were also cases with increased parasympathetic and decreased sympathetic function, indicating that symptoms of autonomic neuropathy are not uniform. They reported that EAT improves dizziness-type symptoms and normalizes autonomic function; EAT suppresses excessive autonomic reflexes and normalizes autonomic function [25]. This paper suggests that it is important to evaluate autonomic function prior to the start of treatment because chronic epipharyngitis presents a variety of autonomic symptoms, and each autonomic symptom is not uniform. It was also believed that improving excessive autonomic reflexes would lead to recovery of autonomic function.

To examine the autonomic nervous system function of patients with abnormal laryngeal sensitivity, Shiraishi applied adrenergic stimulation to the pharyngeal mucosa as local stress and cold stress as systemic stress and compared the changes in blood flow in the pharyngeal mucosa during the application with those in the normal group. In the group of patients with abnormal pharyngeal sensitivity, autonomic nervous system function was impaired, blood flow to the pharyngeal mucosa was reduced, and reactivity to local and systemic stress was decreased. The authors reported that chronic sympathetic nervous system tone reduced the autonomic response to stress [26]. This paper suggests that local and systemic stress increases sympathetic activity and decreases blood flow to the pharyngeal mucosa. Chronic stress was also thought to decrease sympathetic reflexes.

Stimulation of the nasal cavity may induce vagal reflexes via the trigeminal nerve, resulting in bradycardia and hypotension, which may induce syncope attacks due to transient cerebral blood flow deprivation. Masaki reported that mental stress that stimulates the sympathetic nervous system also induces decreased blood flow and blood pressure [27]. Local stress, systemic stress, and mental stress were all thought to induce sympathetic reflexes, resulting in decreased blood flow and blood pressure.

Kusuyama examined the relationship between nasopharyngitis and voice disorders and reported that the sympathetic nervous system stimulation state caused by epipharyngitis is one of the causes of voice disorders. He reported that the stimulating state of the sympathetic nervous system causes laryngeal lubrication disorder and resonance disorder and that the voice disorder can be treated by improving the sympathetic stimulating state with EAT [28]. The sympathetic nervous system is also thought to influence the activity of the mucociliary glands, which may be the cause of voice disorders.

Headache and sore throat are symptoms of chronic epipharyngitis, and EAT has been found to be effective in the treatment of headaches and sore throat. Cluster headaches, Vidian neuralgia, and Sluder's neuralgia are classified as trigeminal autonomic cephalalgias (TACs). These disorders are thought to cause headaches and other symptoms due to activation of parasympathetic reflexes by trigeminal nerve excitation [29]. Puig et al. reported that a pterygopalatine ganglion block (SPG-block) improves symptoms of TACs [30]. Since vagus nerve stimulation suppresses trigeminal parasympathetic nerve reflexes, the pterygopalatine ganglion electric stimulation therapy (SPG-Stim), in which an electric device is implanted in the SPG for electrical stimulation, has been clinically applied to relieve symptoms of TACs [31]. These reports suggest that stimulation or inhibition of trigeminal nerve activity may be useful as a treatment for TACs.

Tanaka reported intranasal sphenopalatine ganglion stimulation (INSPGS) and discussed its mechanism of action [32]. The pterygopalatine ganglion (SPG) is a branch of the second trigeminal nerve and receives both parasympathetic and sympathetic fibers. Rubbing the epipharyngeal mucosa near the nasopharyngeal canopy with EAT stimulates afferents in the SPG innervated area. The author reported that EAT may express effects similar to those of INSPGS and control symptoms such as headache.

Ito used heart rate variability analysis to clarify the autonomic reflex patterns induced by EAT and discussed the mechanism of the immediate effect of EAT. EAT has both sympathomimetic and parasympathomimetic effects, but the response depends on the timing and site of stimulation. During nasa abrasion, parasympathetic stimulation induces a vagal reflex, resulting in a decrease in heart rate. During oral rubbing, sympathetic and parasympathetic nerves are stimulated to elicit the pharyngeal reflex (gag reflex), but the sympathetic and parasympathetic nerves move antagonistically, and their activity is time-lagged and thus works in a balance-regulating manner. The authors reported that the opposing inhibitory and excitatory inputs may facilitate reflexive control and activate autonomic reflex function [33].

Stellate ganglion block (SGB) is a treatment method to suppress or stimulate the autonomic nervous system, which temporarily blocks sympathetic nervous activity to favor parasympathetic activity. Goto reported that autonomic nervous system activity fluctuates due to a rebound phenomenon after functional blockade with SGB [34,35]. SGB repetitive stimulation therapy, which shakes the autonomic nervous system, has an autonomic balancing effect on the sympathetic and parasympathetic nervous systems and activates self-repair functions. There is a reflex mechanism whereby reflex control is facilitated by the simultaneous action of opposing inhibitory and excitatory inputs [36]. When the pharyngeal reflex is elicited, the reflex center neurons are activated simultaneously, followed by a refractory period. Sympathetic and parasympathetic activities may be reset and return to their original state of balance. The mechanism of the therapeutic effect of EAT on the autonomic nervous system may be that the conflicting excitatory and inhibitory input stimuli from simultaneous stimulation of the sympathetic and parasympathetic nervous systems may activate the autonomic reflex function.

The baroreceptor reflex (BR) regulates blood pressure to keep it as constant as possible. When blood pressure rises sharply, sympathetic activity is decreased and parasympathetic activity is increased, thereby decreasing heart rate and vascular resistance and lowering blood pressure to its original level. When blood pressure falls rapidly, it increases sympathetic activity and decreases parasympathetic activity, thereby increasing heart rate and vascular resistance and raising blood pressure to its original value [37].

The active standing (AS) test elicits sympathetic and parasympathetic autonomic reflexes under the influence of central excitatory nerves and baroreceptor signals. The initial orthostatic load causes retention of blood in the veins of the lower extremities and trunk under the influence of gravity. Then, as blood returns to the heart from the lower extremity veins decreases, cardiac output decreases and blood pressure falls. In the normal state, sympathetic activity is quickly increased and parasympathetic activity is quickly suppressed, resulting in an autonomic reflex that increases heart rate and contractility, constricts blood vessels, and maintains blood pressure. Furthermore, continued standing increases circulating blood volume by activation of the renin-angiotensin-aldosterone system and secretion of antidiuretic hormone, and the autonomic reflex suppresses sympathetic nerve activity and increases parasympathetic nerve activity, thereby increasing heart rate, contractility, and vasoconstriction to calm the heart and maintain a blood pressure suitable for standing. This function is to maintain the blood pressure suitable for a standing position. By looking at the blood pressure and heart rate fluctuations in this AS test, the BR function can be captured [38].

Hirobumi performed the AS test and also examined the effect of EAT on BR over time. The results showed that EAT suppressed parasympathetic nerve activity as a temporal effect and tended to suppress blood pressure fluctuation during the AS test. In other words, EAT activates BR, suppresses blood pressure variability, and normalizes autonomic reflex activity [39]. The stabilizing effect of EAT on blood pressure variability has been reported in patients with OD, postural orthostatic tachycardia syndrome, and myalgic encephalomyelitis/chronic fatigue syndrome (ME/CFS).

Kawada analyzed the static and dynamic characteristics of the arterial baroreceptor reflex and pointed out that the autonomic regulatory system responds differently in normal and pathological conditions [40]. Hirobumi compared the AS test between the group with improved autonomic symptoms and the group with unchanged autonomic symptoms after EAT. Compared to the unchanged group, the blood pressure variability in the improved group decreased, indicating that the BR was functioning normally. It was reported that the BR response differs between normal and pathological states of autonomic function [40].

In the reflex arches of the EAT, afferent information from nasal rub stimuli is transmitted from the cribriform nerve (first branch of the trigeminal nerve), the posterior nasal nerve (second branch of the trigeminal nerve), and the pharyngeal plexus (glossopharyngeal nerve, vagus nerve, and cervical sympathetic nerve) to the nucleus of the solitary bundle, the trigeminal spinal tract nucleus, the superior salivary nucleus, and the nucleus of the blue spot. Afferent information triggers autonomic responses via vasomotor reflexes and influences sympathetic and parasympathetic nervous activity. Afferent information from oral abrasion stimulates the glossopharyngeal nerve and sympathetic nerves and is transmitted to the nucleus of suspicion from the nucleus of the solitary bundle and trigeminal spinal tract, and output to the vagal motor branch via the pharyngeal plexus, causing the pharyngeal reflex (pharyngeal reflex) and the gag-reflex (strangulation reflex) [41]. The EAT reflex arch is thought to modulate autonomic functions of the upper airway and upper gastrointestinal tract in a reflex-defensive manner.

EAT stimulation of trigeminal afferents inputs to the trigeminal tract nucleus, with the intermediate subnucleus involved in the vasodilatory response and the caudal subnucleus in the vasoconstrictor response. Differences in the opposing circulatory responses of bucking or boosting pressure are observed among stimulus conditions, animal species, and individuals [42]. The EAT is thought to regulate BR via vasomotor and supraventricular centers.

Porges proposed the polyvagal theory, which states that the vagus nerve, which represents the parasympathetic nervous system, is composed of two types of nervous systems with different phylogenetic origins, and that the autonomic response to stress involves a hierarchical response of two types of vagal nerves, the ventral and dorsal vagal systems, and three types of sympathetic nervous systems. He proposed the multiple vagal theory (polyvagal theory) [9]. When the epipharyngeal mucosa is stimulated nasally, the ventral vagal system is stimulated to suppress the heart rate, but when the epipharyngeal mucosa is stimulated orally, the sympathetic and dorsal vagal control mechanisms are simultaneously involved, and a pharyngeal reflex is thought to appear.

ME/CFS is thought to be a disorder of homeostasis due to an overstimulated or exhausted autonomic nervous system. The vagus nervous system is unable to modulate the sympathetic nervous system's response to stress in an inhibitory manner, i.e., vagal homeostasis is impaired, which may be one of the pathogenesis of ME/CFS. In chronic epipharyngitis, the sympathetic nervous system is continuously stimulated and becomes hyper- or hypoactive, the vasomotor reflex is suppressed, and the BR does not function normally, resulting in exaggerated blood pressure variability. Autonomic regulation is deficient, and an exaggerated response of the dorsal vagal system may lead to the development of pathological vagal reflexes and Reilly's phenomenon [43]. With repeated EAT, chronic epipharyngitis improves over time, local, general, and psychological stress is relieved, and the hyperactive and hypoactive states of sympathetic nervous activity are improved. In addition, as chronic epipharyngitis improves, parasympathetic activity is normalized, the sympathetic reflexes are regulated, and the vasomotor reflex and BR are thought to be normalized.

Effects of EAT on the endocrine system

Yamada demonstrated that EAT promotes blood cortisol (11-OHCS) secretion and reported that it is effective for allergic rhinitis and collagen diseases [44]. EAT is thought to affect the hypothalamus-pituitary-adrenocortical system (HPA system) and to be involved in the endocrine system.

Ito examined the effects of psychological and physical stress on salivary amylase activity by EAT and reported that salivary amylase activity increased by EAT. In addition, it was reported that there was a sex difference in the response. Since salivary amylase activity is affected by sympathetic nervous activity, EAT may stimulate sympathetic nervous system activity, and EAT affects the sympathetic-adrenal medulla (SAM) system.

In response to acute stress, the SAM system, which has a short response latency, is first activated to maintain the internal environment by maintaining dynamic homeostasis. In response to chronic stress, the HPA system is activated to maintain homeostasis. Although EAT stimulation initially acts as an acute stress, repeated and continuous EAT stimulation is thought to induce an allostatic response in the body, and the SAM system repeatedly adapts to the stress. The SAM system may be responding adaptively so that it can respond more quickly to stressful stimuli [45].

Influence of EAT on the immune system

Hotta proposed tonsillectomy combined with steroid pulse therapy as a treatment for IgA nephropathy. He reported the therapeutic effect of EAT on the immune system [11]. Mogitate reported the efficacy of EAT in IgA nephropathy [46].

Hotta also reported that EAT has a therapeutic effect on Human Papillomavirus Vaccine-associated Neuroimmunopathic Syndrome (HANS) [47], an adverse reaction to the cervical cancer vaccine. HANS is often associated with chronic epipharyngitis, which improves with EAT. EAT also improves limbic system dysfunction such as chronic fatigue and brain fog, suggesting that there may be an epipharynx-limbic system axis interaction between the nasopharynx and limbic system [48]. 

Mogitate studied the changes in CD4 and CD8 profiles of epipharyngeal mucosa cells before and after EAT treatment. CD4(+) T cells are significantly increased in patients with chronic epipharyngitis, and CD4(+) T cells are significantly decreased with EAT treatment. It was reported that CD4(+) T cells play an important role in the persistent inflammation of chronic epipharyngitis [13]. A link between chronic epipharyngitis and the immune system was demonstrated.

Mogitate also found that EAT decreased fractional exhaled nitric oxide (FeNO) levels [49], suggesting that EAT causes squamous epithelialization of the epipharyngeal mucosa lineage epithelium and lowers FeNO. The decrease in FeNO may suppress type 2 inflammation such as asthma, indicating a link between EAT and the immune system.

Nishi reported that the expression of IL-6 and TNFα in the epipharyngeal mucosa is increased in chronic epipharyngitis and that EAT suppresses their production, revealing an immunohistological anti-inflammatory effect of EAT [12,50].

Overproduction of IL-6 is implicated in the pathogenesis of many autoimmune and inflammatory diseases. Since IL-6 attenuates baroreceptor reflex function in the medullary solitary bundle nucleus [51], it is possible that BR is reduced in cases of chronic epipharyngitis. EAT may suppress IL-6 and activate BR by immunological effect

Tracey reported an inflammatory reflex in which the immune system is regulated by stimulation of the nervous system. Vagus nerve stimulation suppresses the production of inflammatory cytokines [52]. Electrical stimulation of the afferent vagus nerve enhances the function of inhibitory systems such as GABA, and vagus nerve stimulation (VNS) therapy has been applied clinically to treat autoimmune diseases, depression, and intractable epilepsy. Transcutaneous VNS also enhances brain plasticity and has been clinically applied for stroke rehabilitation. Afferent vagal stimulation affects both the nervous and immune systems, increases cranial nerve plasticity, and improves autonomic function [53]. EAT may have VNS-like effects.

Atsumi et al. reported that non-immune cells are induced by IL-6 signals. They named this mechanism of inflammation formation centered on non-immune cells the “inflammation amplifier” [54]. In the central nervous system, the blood-brain barrier restricts the entry of immune cells and macromolecules from the blood, Sabharwal reported the gateway reflex, in which sympathetic nerve stimulation activates β1-adrenergic receptors, induces inflammation, and opens the blood-brain barrier at specific vascular sites [55]. Arima investigated the molecular mechanisms of the effects of pain stress, mental stress, electrical stimulation, and sleep disturbance on chronic inflammatory pathology, and found that activation of sensory and sympathetic nerves is an important factor in the regulation of inflammation in the central nervous system [56]. Chronic epipharyngitis induces inflammation in specific areas of the CNS via the autonomic nervous system and the immune system, and EAT may regulate inflammation in these areas.

Various symptoms such as malaise, dyspnea, brain fog, and autonomic neuropathy develop in post-neoplastic coronary syndrome (Long COVID: LC), and it has been reported that these may be caused by vagus nerve damage. Chronic stimulation of the vagus nerve induces encephalitis, which leads to the development of autonomic neuropathy symptoms [57]. The efficacy of EAT on LC has been reported [58-59], and it is possible that EAT exerts its therapeutic effect on LC by suppressing the increase in parasympathetic activity caused by vagal inflammation [43]. Vagus nerve stimulation can induce or control inflammation. It is important to determine which mechanism is predominant when performing EAT and to apply it to treatment.

Interaction of EAT with the effects of stimulation of the autonomic nervous system, immune system, and endocrine system

In humans, the autonomic nervous system, endocrine system, and immune system work together to maintain homeostasis in response to environmental changes inside and outside the body. Among these regulatory systems, the endocrine and immune systems are systemic and take time to exert their functions, whereas the nervous system's regulation is instantaneous, rapid, and strongly localized. The core of the nervous system's regulation is the autonomic nervous system, which is anatomically divided into two major systems: the sympathetic nervous system and the parasympathetic nervous system. The organism is built on the coordination and interaction of these systems, each influencing the other. The keynote of autonomic nervous system activity is parasympathetic activity, while sympathetic nervous system function is reflexive activity [60].

Stimulation of the airway mucosa causes a cough reflex with the glossopharyngeal nerve and vagus nerve as afferent pathways and stimulation of the nasal mucosa causes a sneeze reflex with the trigeminal nerve as an afferent pathway. These reflexes are defensive reflexes to expel foreign substances that have invaded the airway, and sympathetic nerve activity becomes active when these defensive reflexes appear. Their activity is modulated by parasympathetic activity. EAT stimulation is thought to basically trigger the defensive reflexes.

**Figure 1:**
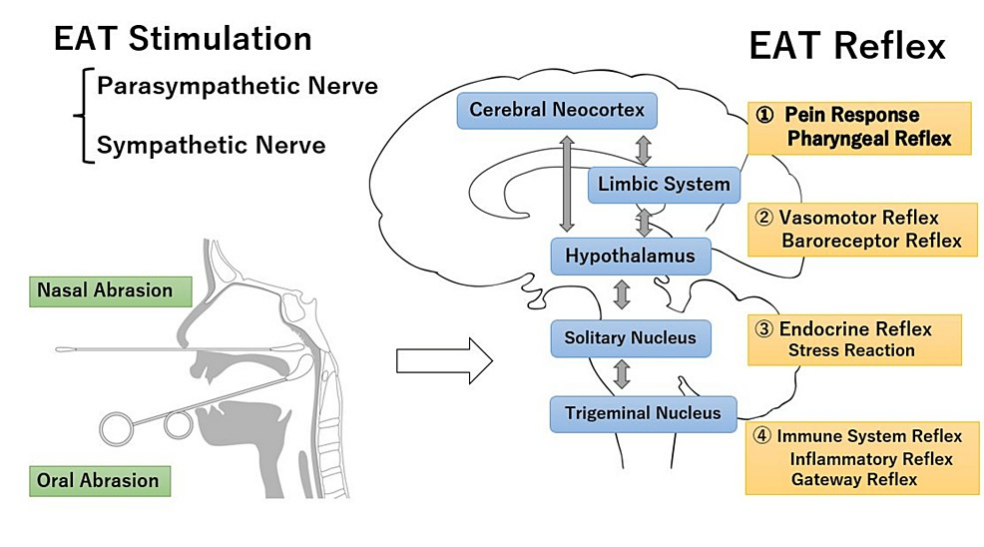
Summary of the EAT reflex EAT is delivered by intranasal and oral abrasion and stimulates sympathetic and parasympathetic nerves in the nasal cavity and epipharyngeal mucosa. EAT elicits pain responses and defensive reflexes such as the pharyngeal reflex. It affects vasomotor and baroreceptor reflexes in the cardiocirculatory autonomic nervous system and has therapeutic effects on chronic epipharyngitis. It also affects endocrine system reflexes and expresses stress responses. The immune system may induce inflammatory and gateway reflexes, affecting various organs of the body and the central nervous system. The autonomic nervous system, endocrine system, and immune system influence each other. EAT: Epipharyngeal abrasive therapy Image Credit: Author

Furthermore, EAT stimulation stimulates the neuronal network of the brainstem, which consists of the swallowing, vomiting, respiratory, and circulatory centers, and also influences endocrine reflexes via the hypothalamus and pituitary gland and stress responses via the limbic system. The EAT reflex is an integration of several autonomic reflexes and is hierarchically regulated. The immune system, endocrine system, and autonomic nervous system are thought to interact with each other to produce therapeutic effects (Figure 1) [61].

This paper is a literature review focusing primarily on the effects of EAT on the autonomic nervous system. In individual inflammatory conditions, it is important to determine which mechanism is predominantly involved and to utilize it in treatment. Understanding the effects of EAT on the autonomic nervous system will lead to a better understanding of the mechanism of action of EAT and will be an important guide in determining a treatment strategy for chronic epipharyngitis.

## Conclusions

Autonomic neuropathy symptoms are caused by chronic stimulation of the autonomic nervous system and persistent irritation due to inflammation-related factors, stressors, and other factors. There is also a decrease in autonomic nervous system function due to circulatory disturbance of the autonomic nerve center.

EAT has the effect of improving local inflammation of the mucous membranes and resolving autonomic nervous system stimulation, as well as restoring venous and lymphatic system function and stimulating the autonomic nerve center. EAT stimulation also induces various autonomic reflexes and activates autonomic functions. Specifically, EAT normalizes the vasomotor and baroreceptor reflexes and controls blood pressure variability. The EAT reflex is an integration of several autonomic reflexes and is hierarchically regulated. The immune system, endocrine system, and autonomic nervous system interact with each other to produce therapeutic effects.
